# P-1792. Bridging Genomics and Clinical Medicine: RSVrecon Enhances RSV Surveillance with Automated Genotyping and Clinical-important Mutation Reporting

**DOI:** 10.1093/ofid/ofaf695.1961

**Published:** 2026-01-11

**Authors:** Lei Li, Haidong Yi, Jessica Brazelton, Richard Webby, Randall Hayden, Gang Wu, Diego R Hijano

**Affiliations:** St. Jude Children's Research Hospital, Memphis, TN; St. Jude Children's Research Hospital, Memphis, TN; St. Jude Children's Research Hospital, Memphis, TN; St. Jude Children's Research Hospital, Memphis, TN; St. Jude Children's Research Hospital, Memphis, TN; St. Jude Children's Research Hospital, Memphis, TN; St. Jude Children's Research Hospital, Memphis, TN

## Abstract

**Background:**

Respiratory Syncytial Virus (RSV) causes significant respiratory infections, particularly in young children and elderly adults. Genetic variations in the fusion (F) protein can reduce the efficacy of vaccination and monoclonal antibody treatments, emphasizing the need for genomic surveillance of this virus. Current pipelines for RSV genome assembly focus on sequence reconstruction but often lack features for detecting genotypes, clinically relevant mutations, or presenting results in formats that are suitable for clinical researchers.

**Figure 1.**
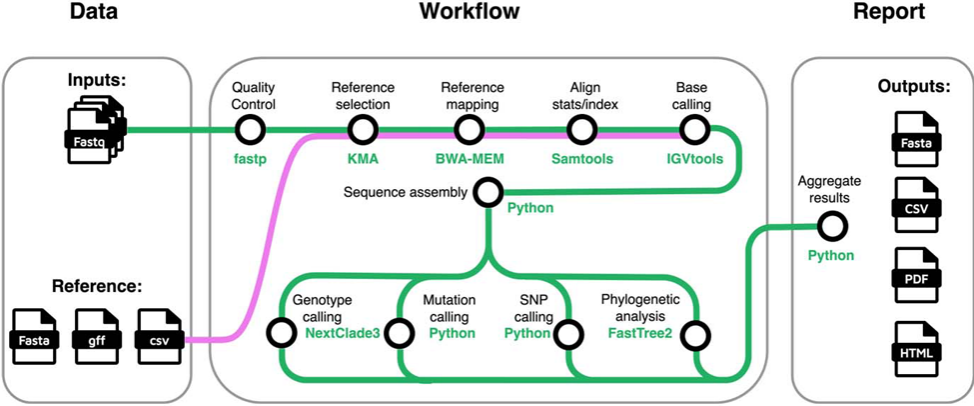
Overview of the RSVrecon pipeline workflow. The pipeline uses raw sequences (paired FASTQ) as inputs from users. It then sequentially processes the data. Outputs include consensus sequences (FASTA) and aggregated results in multiple formats (CSV, PDF, HTML). The workflow is implemented in NextFlow, enabling parallelized execution and containerized reproducibility. Green paths represent the data flow of sequencing data through the pipeline, whereas purple paths indicate the data flow of the pre-built reference datasets.

**Figure 2.**
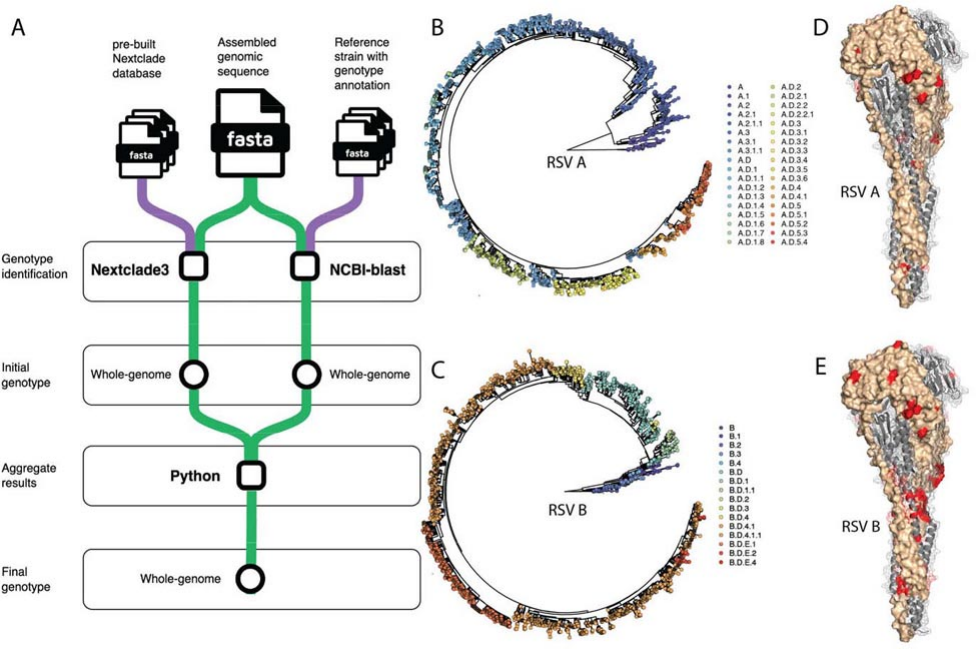
Automated genotype identification and screening of clinically important mutations in the F protein. A: Workflow of genotype identification. Whole-genome-based genotypes are inferred using two approaches independently. Results from both approaches are aggregated for a final call of genotype. B, C: Genotypes and genetic lineages of RSV A and RSV B are defined using the most recent dataset. Phylogenetic trees are based on whole-genome sequences. Trees and metadata are downloaded from Nextstrain (https://nextstrain.org/). D, E: Clinically relevant mutations in F protein for RSV A and RSV B, visualized on the structure of F protein in the post-fusion conformation (PDB ID: 3RRR).

**Methods:**

We developed a reference-based computational pipeline for comprehensive RSV genomic analysis, integrating quality control, reference mapping, consensus calling, phylogenetic analysis, and genotype assignment using cutting-edge bioinformatics tools. Its modular design, powered by Nextflow's modern framework, ensures easy installation, scalability, and cross-platform compatibility.

**Figure 3.**
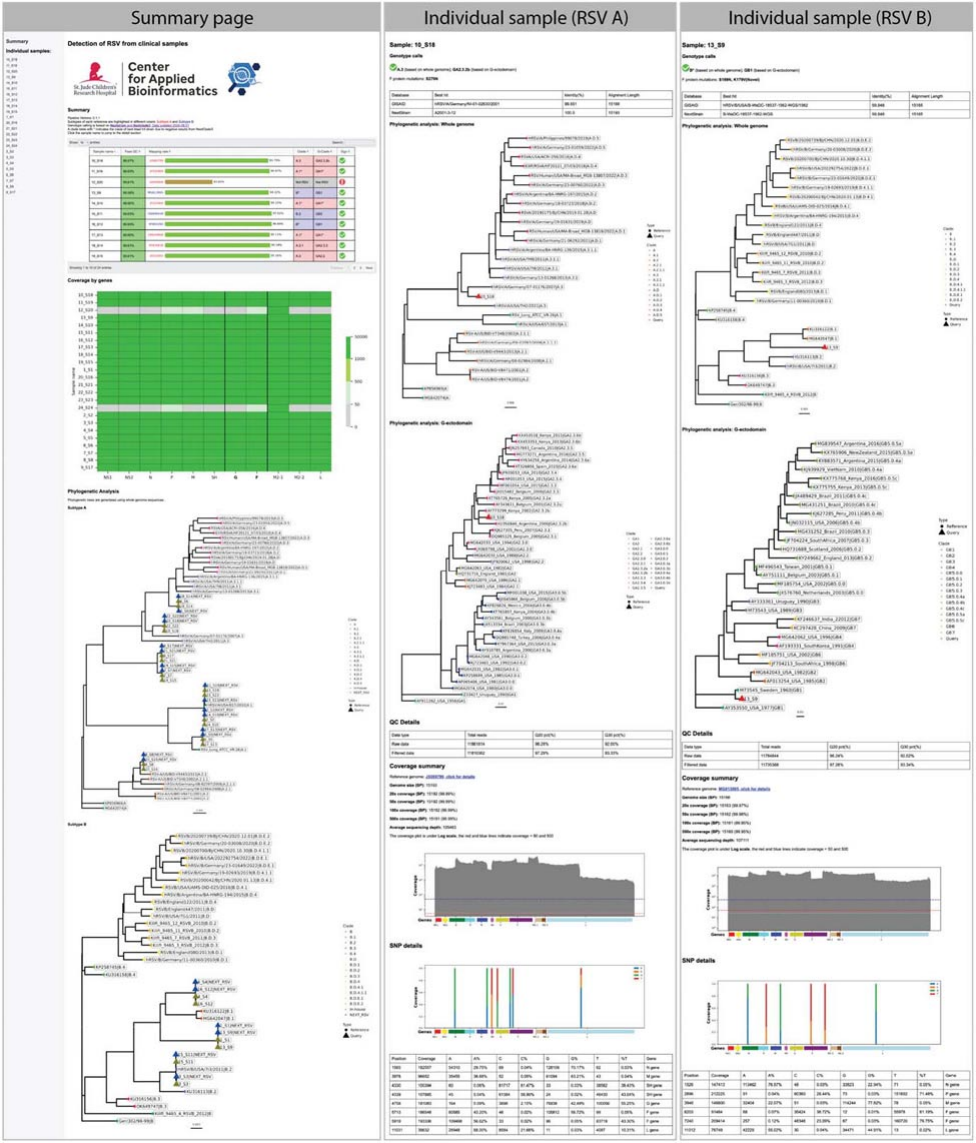
Accessible and User-Friendly Outputs of RSVrecon. The pipeline generates two levels of comprehensive reports: (1) a batch-level summary displaying QC metrics, mapping quality, subtypes, genotypes, gene coverage profiles, and phylogenetic relationships across all samples, and (2) detailed sample-specific reports featuring assembly quality metrics, genotype characterization, F protein mutations, best-matching reference sequences, whole-genome and G-gene phylogenetic placement, coverage statistics, and SNP annotations.

**Table 1.**
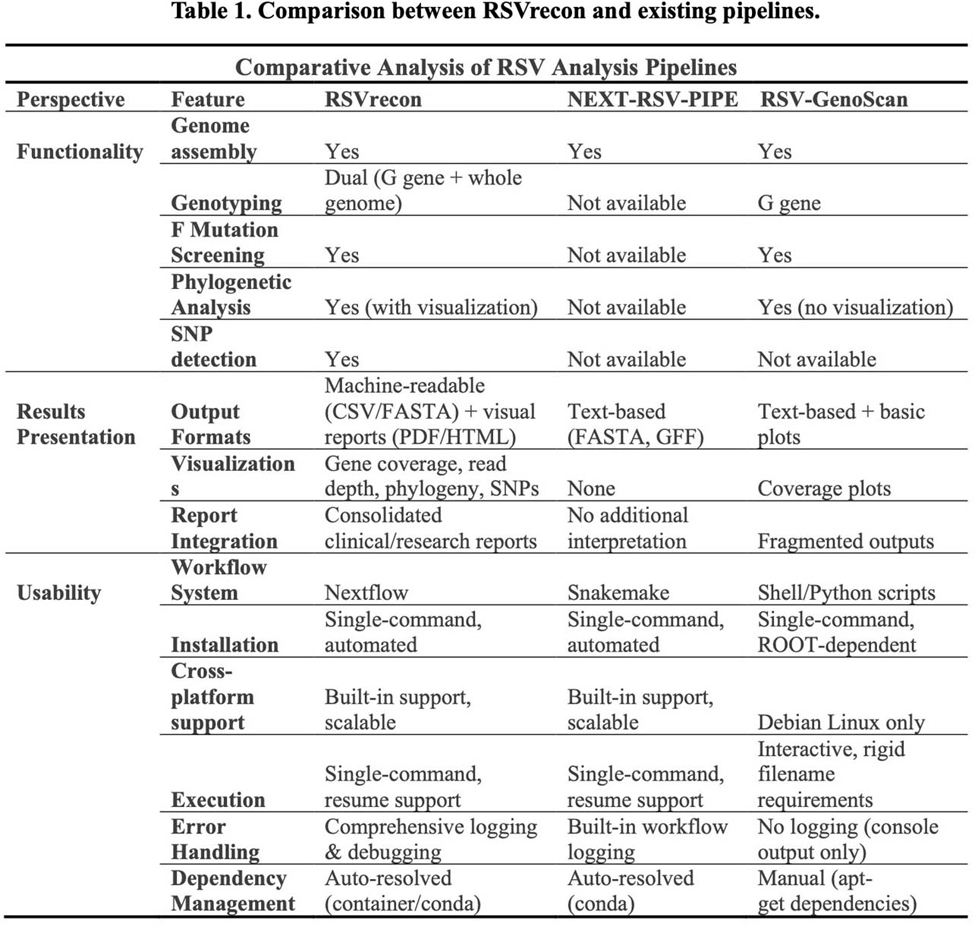
Comparison between RSVrecon and existing pipelines.

**Results:**

RSVrecon processes raw FASTQ files into annotated variant reports and delivers results in multiple formats (CSV, PDF, HTML) tailored to diverse end users. A key innovation of RSVrecon is its integrated detection of clinically critical features, including genotype classification and F protein mutation calling, capabilities that are absent in most analytical pipelines. Benchmarking against existing pipelines using clinical datasets revealed that RSVrecon achieves comparable genomic assembly accuracy while excelling in three key dimensions: (1) expanded functional capabilities, (2) intuitive biological interpretation of results, and (3) superior user experience and accessibility.

**Conclusion:**

By seamlessly translating RSV genomic data into clinically meaningful information, RSVrecon empowers research, with the potential to guide clinical care decisions and strengthens surveillance systems. These features make RSVrecon an excellent solution for RSV surveillance and research.

**Disclosures:**

Randall Hayden, MD, Abbott: Board Member|Abbott: Serving on the advisory board|Cepheid: Board Member|Cepheid: Serving on the advisory board|Roche Diagnostics: Advisor/Consultant|Roche Diagnostics: Board Member|Roche Diagnostics: Serving on the advisory board

